# Supplementary material for: 3D histology of human heart-forming organoids by X-ray phase-contrast tomography
Source: Commun Biol. 2025 Oct 1;8:1411. doi: 10.1038/s42003-025-08876-1 (PMC12488953; doi:10.1038/s42003-025-08876-1)
Supplement: Supplementary file 2 — Supplementary Information [file 42003_2025_8876_MOESM2_ESM.pdf]

Supplementary Information: 3D histology of human heart-forming organoids by X-ray phase-contrast tomography

Karlo Komorowski[1], Jakob Reichmann[2], Lika Drakhlis[3], Robert Zweigerdt[3], and Tim Salditt[2]

[1]Department of Radiation Protection and Medical Physics, Hannover Medical School, Hannover, Germany
[2]Institute for X-Ray Physics, University of Göttingen, Göttingen, Germany
[3]Leibniz Research Laboratories for Biotechnology and Artificial Organs (LEBAO), Department of Cardiothoracic, Transplantation and Vascular Surgery (HTTG), REBIRTH-Research Center for Translational Regenerative Medicine, Hannover Medical School, Hannover, Germany

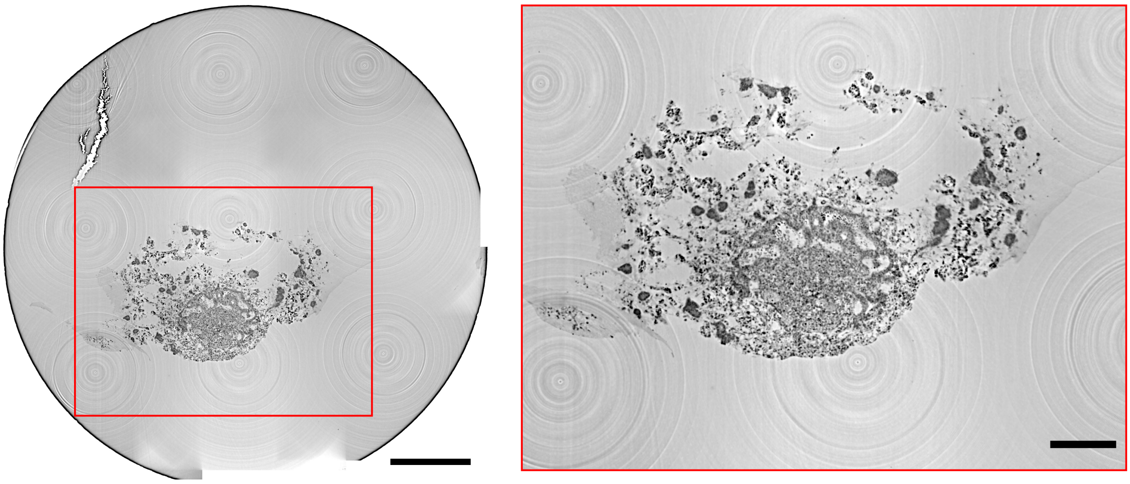

Supplementary Fig. 1. Stitched tomograms. Reconstructed slice through a stitched volume of nine datasets obtained by the SR-PB setup of a FFPE HFO (HFO Sample 3) in a 3 mm sized punch biopsy. The resulting FOV is approximately 4×4 mm^2 and the effective voxel size is 1.3 µm after 2×2-binning of the individual datasets. Scalebar is 500 µm (left) and 200 µm (right).

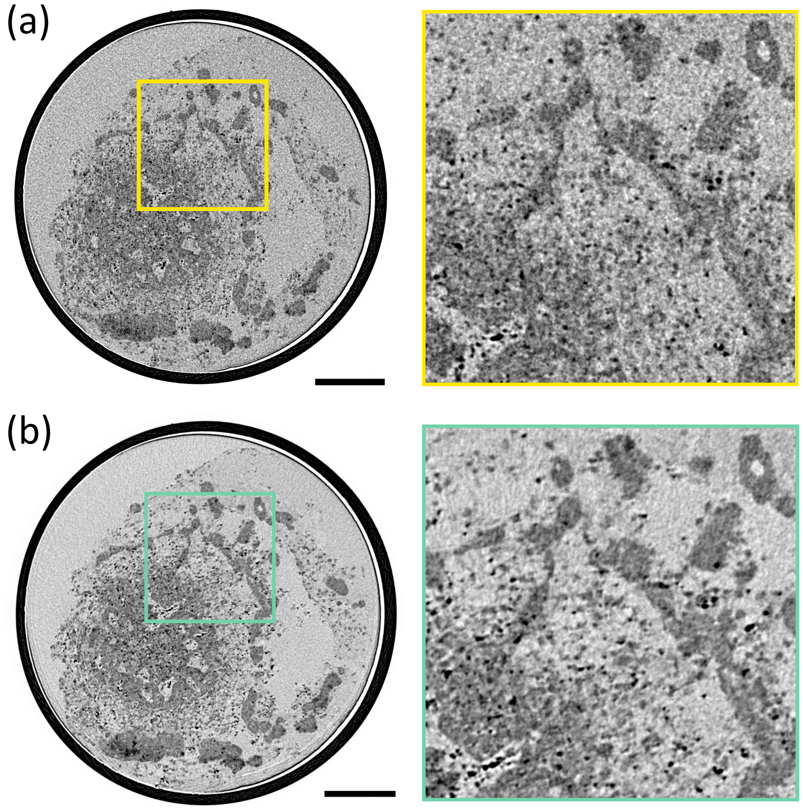

Supplementary Fig. 2. Laboratory μ-CT of HFO Sample 1. Comparison of the image quality of two reconstructed slices at approximately the same position obtained by (a) a tomographic scan with a total acquisition time of 1.75 hours and (b) a long tomographic scan with a total acquisition time of 21.8 hours. Contrast and SNR are significantly higher with the longer scan, but the image quality of the tomographic recording with relatively short acquisition time shows that quick overview scans can be obtained to identify relevant structural details, scalebar = 200 μm.

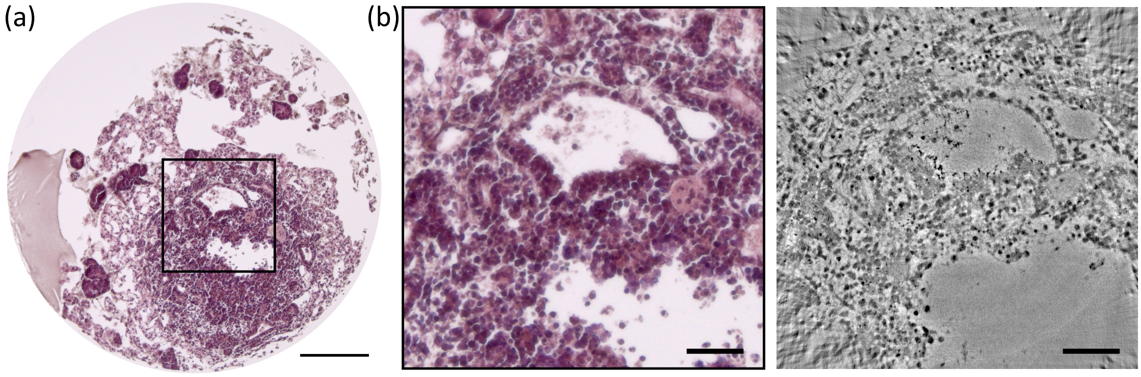

Supplementary Fig. 3. H&E histology of HFO Sample 4. (a) Light microscopy image of an histological slice of an HFO, correlated to (b) a reconstructed virtual slice obtained by the high-resolution SR-CB setup. H&E-staining and sectioning was performed after the tomographic recording. Scalebar is 200 µm in (a) and 50 µm in (b).

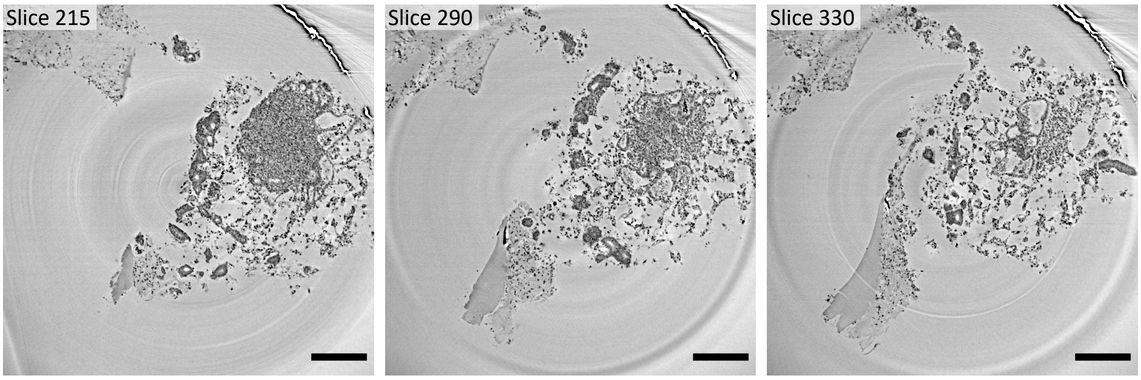

Supplementary Fig. 4. NKX2.5-KO HFO. Series of virtual slices through the reconstructed volume of a NKX2.5 knockout HFO in a 3 mm sized punch biopsy. The tomographic recording was obtained using the SR-PB setup, scalebar = 200 µm. Notably, a reduced adhesion between cardiomyocytes can be observed in the ML.

Estimation of the resolution

The spatial resolution in the tomographic reconstructions for each of the experimental configurations (SR-PB, SR-CB, and laboratory-based µ-CT (Easytom)) is estimated using the half-width-half-maximum (HWHM), which is obtained by fitting line-cuts through edges of cell nuclei to an error-function profile (see Supplementary Fig. 5 and 6). The analysis has been performed with the Fiji software by using the fit model

$$f(x) = a + b \cdot \operatorname{erf}\left(\frac{x-c}{d}\right) \tag{1}$$

with fit parameters a, b, c, d, and error-function

$$\operatorname{erf}(x) = \frac{2}{\sqrt{\pi}} \int_0^x e^{-t^2} \mathrm{d}t \ . \tag{2}$$

The error function can be related to the integrated Gaussian distribution $\Phi(x)$ with the standard deviation σ and the expected value μ by

$$\Phi(x) = \frac{1}{2}\left(1 + \operatorname{erf}\left(\frac{x-\mu}{\sqrt{2}\sigma}\right)\right) \ , \tag{3}$$

so that HWHM $= 2\sqrt{2 \ln 2}\sigma/2$ is calculated with $\sigma = d/\sqrt{2}$ for each line-profile (the results are summarized in Supplementary Tab. 1). Since the steepness of the line-cuts depends on the specific features of the nuclei, the average HWHM was calculated from multiple line profiles. As expected, the highest resolution, i.e. $\mathrm{HWHM}_{\mathrm{mean}} \approx 0.27$ µm, has been achieved by the SR-CB setup. Meanwhile, the reconstructions obtained by the SR-PB and the laboratory µ-CT setup, $\mathrm{HWHM}_{\mathrm{mean}} \approx 0.81$ and 0.88 µm, respectively, demonstrate comparable resolution.

Supplementary Tab. 1. Resolution estimates. Fit parameter d and HWHM for each line profile and for each experimental setup. The line profiles are numbered according to those presented in Fig. 5 and 6. The mean value of HWHM ($HWHM_{mean}$) and the ratio $HWHM_{mean}/px_{eff}$ with the effective pixel size px_{eff} (0.127 µm for SR-CB, 0.65 µm for SR-PB and 0.72 µm Easytom laboratory µ-CT) are given for each tomographic configuration. In the case of SR-PB, line-profile 4 was not considered in $HWHM_{mean}$ since HWHM $<$ px_{eff}.

Setup	Line profile	d (µm)	HWHM (µm)	$HWHM_{mean}$ (µm)	$HWHM_{mean}/px_{eff}$
SR-CB	1	2.77	0.295	0.268	2.1
	2	2.59	0.275		
	3	1.7	0.18		
	4	2.51	0.265		
	5	3.06	0.325		
SR-PB	1	1.51	0.815	0.814	1.3
	2	1.21	0.655		
	3	1.74	0.94		
	4	0.94	0.51		
	5	1.56	0.845		
Easytom	1	1.37	0.82	0.882	1.2
	2	1.41	0.84		
	3	1.38	0.825		
	4	1.61	0.965		
	5	1.6	0.955		

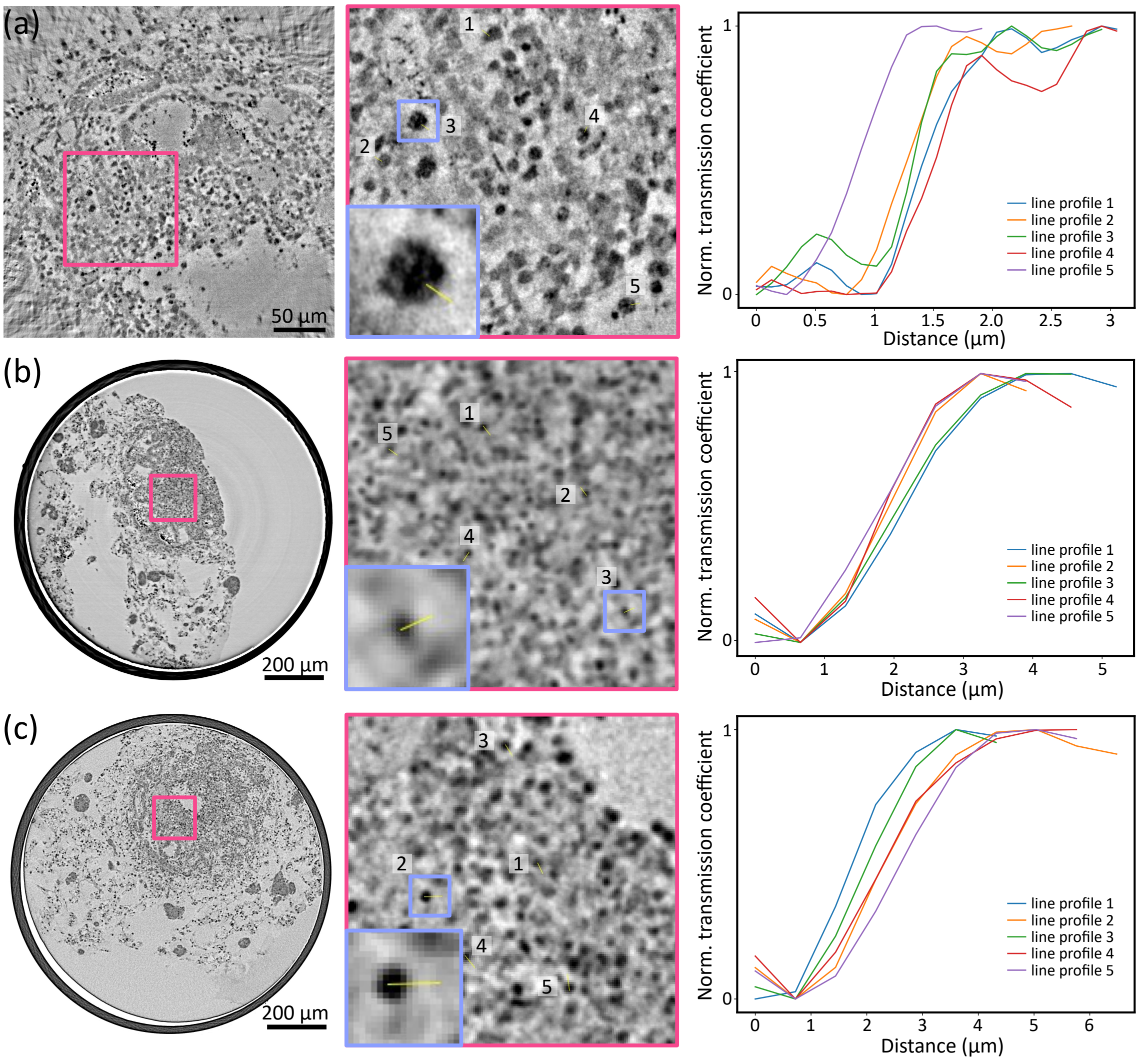

Supplementary Fig. 5. Resolution estimates: Line-cuts through edges of cell nuclei. Line-cuts through edges of cell nuclei are shown for (a) SR-CB, (b) SR-PB, and (c) laboratory µ-CT (Easytom) data for representative slices through the tomographic reconstruction. Five line profiles with a width of 1 pixel are considered for each data set (indicated by the corresponding number in the magnified view, shown by the pink rectangles), and are plotted as a function of distance (on the right). For better visibility, an example line cut through an edge of a cell nucleus is shown in the magnified view within purple rectangles.

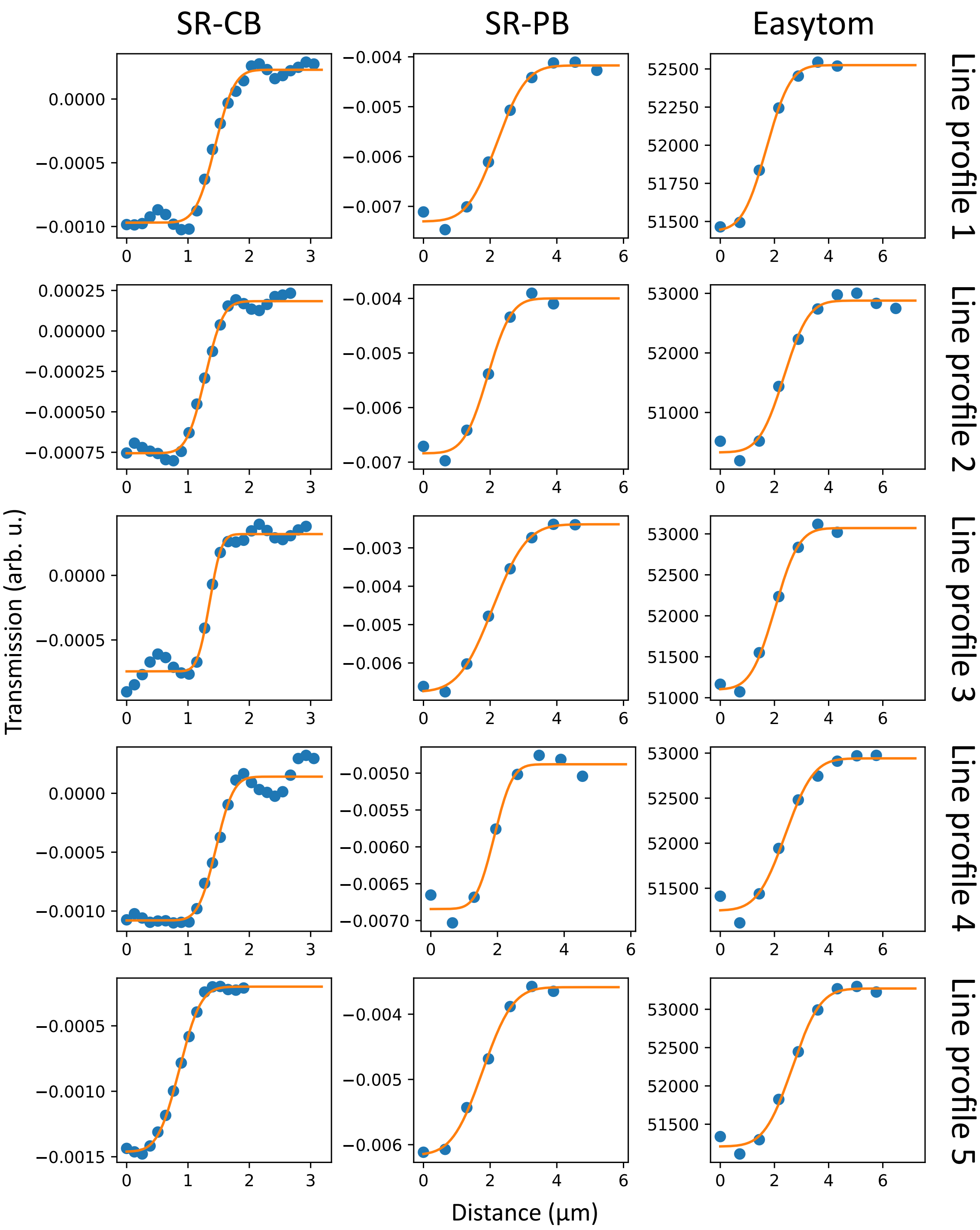

Supplementary Fig. 6. Resolution estimates: Line profiles and fits. Line profiles (blue dots) and the corresponding fits (orange lines) are shown for each of the different tomographic configurations, namely the SR-CB, the SR-PB and the laboraroty µ-CT (Easytom) setup.

Contrast-to-noise ratio

The contrast-to-noise ratio (CNR) was estimated for each of the three tomographic configurations, i.e. SR-CB, SR-PB and laboratory-based µ-CT (Easytom setup), by

$$\mathrm{CNR} = \frac{|\mu_{\mathrm{A}} - \mu_{\mathrm{B}}|}{\sigma} \ , \tag{4}$$

where μ_{A} and μ_{B} are the mean signals in two regions A and B, respectively, and σ is the image noise. To this end, contrast was measured between an endodermal island and the surrounding paraffin-background, see regions-of-interest (ROI) A and B in Supplementary Fig. 7 (correspondingly the mean signals $\mu_{\mathrm{A,B}}$), respectively. Further, image noise was measured by the standard deviation $\sigma_{\mathrm{B}} = \sigma$ of the paraffin signal in ROI B. The SR-PB setup achieves the highest CNR value (CNR = 22.3), followed by the laboratory-based µ-CT (Easytom) setup (CNR = 5.7) and the SR-CB setup (CNR = 3.4), which has the lowest CNR value.

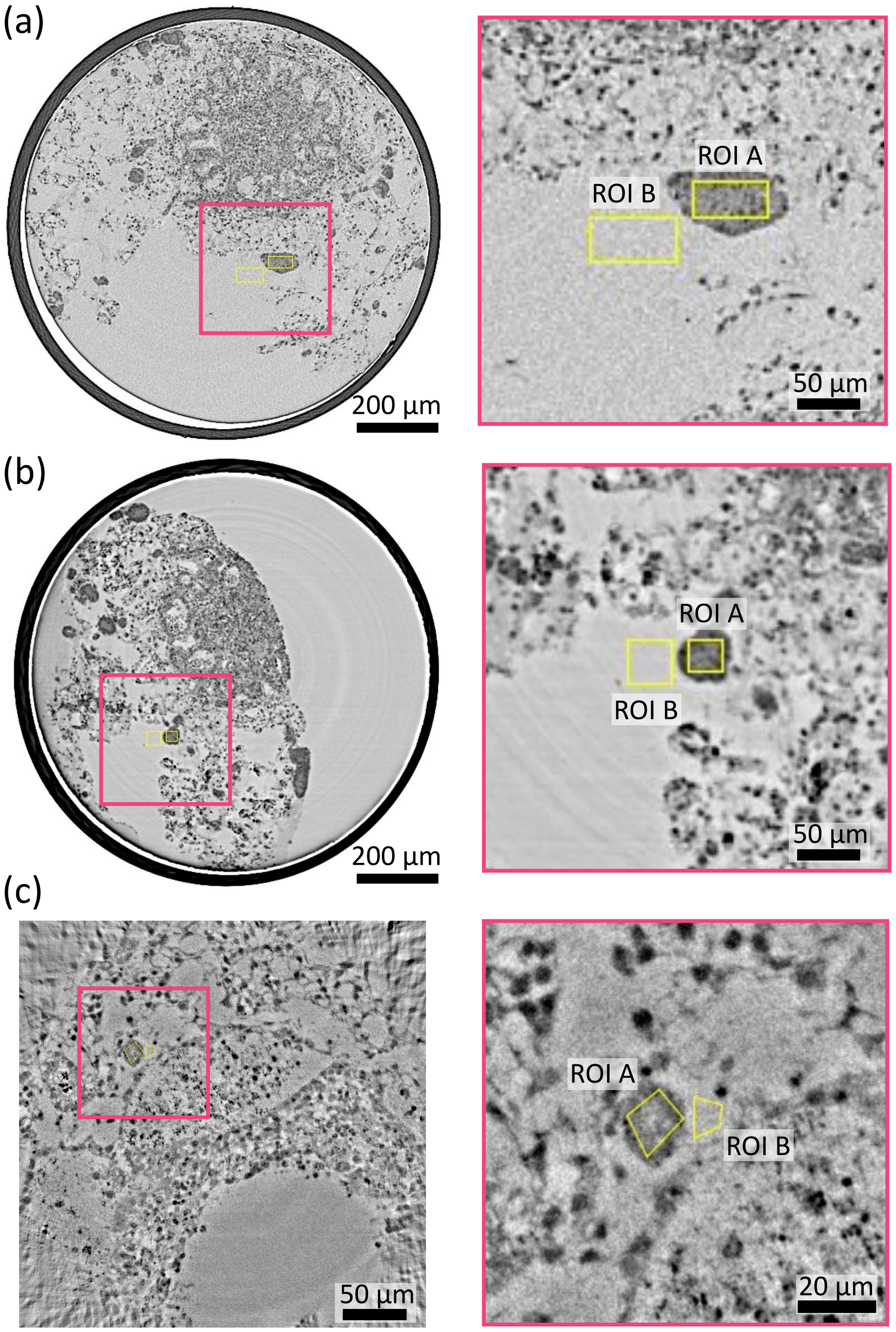

Supplementary Fig. 7. Estimation of the CNR. Representative slices through the tomographic reconstructions from (a) laboratory µ-CT (Easytom), (b) SR-PB and (c) SR-CB datasets. ROI A and B used to measure the mean signal and the standard deviation of the mean signal in an endodermal cavity and in the surrounding paraffin, respectively, are indicated by yellow quadrangles. The resulting CNR values are 5.7 for (a), 22.3 for (b) and 3.4 for (c).
