# Supplementary material for: 3D histology of human heart-forming organoids by X-ray phase-contrast tomography
Source: Commun Biol. 2025 Oct 1;8:1411. doi: 10.1038/s42003-025-08876-1 (PMC12488953; doi:10.1038/s42003-025-08876-1)
Supplement: Supplementary file 3 — Description of Additional Supplementary Files [file 42003_2025_8876_MOESM3_ESM.pdf]

Description of Additional Supplementary Files

File name- Supplementary Data 1

File description – The source data behind the graph in Fig. 6(c) of the manuscript.
